# Extent of inappropriate prescription of artemisinin and anti-malarial injections to febrile outpatients, a cross-sectional analytic survey in the Greater Accra region, Ghana

**DOI:** 10.1186/s12936-019-2967-8

**Published:** 2019-09-27

**Authors:** Harriet Affran Bonful, Adolf Kofi Awua, Martin Adjuik, Doris Tsekpetse, Richard Mawuko Kofi Adanu, Pricillia Awo Nortey, Augustine Ankomah, Kwadwo Ansah Koram

**Affiliations:** 1 Ghana College of Pharmacists, Cantonments, P. O. Box CT 10740, Accra, Ghana; 2 Cellular and Clinical Research Centre, Radiological and Medical Sciences Research Institute, Ghana Atomic Energy Commission, Kwabenya, Accra, Ghana; 3 Department of Epidemiology and Biostatistics, School of Public Health, University of Health and Allied Sciences, PMB 31, Ho, Ghana; 4 Research and Development Division, Ghana Health Service, Accra, Ghana; 5 Department of Population and Family Health, School of Public Health, University of Ghana, Accra, Ghana; 6 Department of Epidemiology and Disease Control, School of Public Health, University of Ghana, Accra, Ghana; 7 Department of Epidemiology, Noguchi Memorial Institute for Medical Research, University of Ghana, Accra, Ghana; 8 Population Council, Yiyiwa Drive, Accra, Ghana

**Keywords:** Febrile, Inappropriate prescription, ACT, Greater Accra, Ghana

## Abstract

**Background:**

Febrile children seen in malaria hypo-endemic settings, such as the Greater Accra region (GAR) of Ghana are more likely to be suffering from a non-malarial febrile illness compared to those seen in hyper-endemic settings. The need for prescribers to rely on malaria test results to guide treatment practices in the GAR is even greater. This study was designed to investigate the factors associated with inappropriate artemisinin-based combination therapy (ACT) prescription.

**Methods:**

A survey was conducted in six health facilities in the region in 2015. Treatment practices for febrile outpatient department (OPD) patients were obtained from their records. Prescribers were interviewed and availability of malaria commodities were assessed. The primary outcome was the proportion of patients prescribed ACT inappropriately. Independent variables included patient age and access to care, prescriber factors (professional category, work experience, access to guidelines, exposure to training). Data were analysed using Stata at 95% CI (α-value of 0.05). Frequencies and means were used to describe the characteristics of patients and prescribers. To identify the predictors of inappropriate ACT prescription, regression analyses were performed accounting for clustering.

**Results:**

Overall, 2519 febrile OPD records were analysed; 45.6% (n = 1149) were younger than 5 years. Only 40.0% of patients were tested. The proportion of patients who were prescribed ACT inappropriately was 76.4% (n = 791 of 1036). Of these 791 patients, 141 (17.8%) were prescribed anti-malarial injections. Patients seen in facilities with rapid diagnostic tests (RDT) in stock were less likely to be prescribed ACT inappropriately, (AOR: 0.04, 95% CI 0.01–0.14, p < 0.001) compared to those seen in facilities with RDT stock-outs. Prescribers who had been trained on malaria case management within the past year were 4 times more likely to prescribe ACT inappropriately compared to those who had not been trained (AOR: 4.1; 95% CI (1.5–11.6); p < 0.01). Patients seen by prescribers who had been supervised were 8 times more likely to be  prescribed ACT inappropriately.

**Conclusion:**

Inappropriate ACT prescription to OPD febrile cases was high. Training and supervision of health workers appears not to be yielding the desired outcomes. Further research is needed to understand this observation.

## Background

Malaria remains a significant contributor of morbidity in Ghana. Specifically, it was responsible for between 30.9 and 44.0% of all outpatient department (OPD) cases from 2009 to 2016 [1–6], and between January and March 2017, 2.3 million suspected malaria cases, which required treatment [7], were seen in OPDs across the country.

The National Malaria Control Programme (NMCP) of Ghana has promoted the Test, Treat and Track Policy (T3 Policy), since 2010, as recommended by the World Health Organization (WHO) for the management of uncomplicated malaria cases. According to the T3 policy, febrile patients must, as much as possible, be tested for malaria with a rapid diagnostic test kit (RDT) or by microscopy before treatment. Additionally, the policy encourages the screening of febrile patients for other non-malarial illnesses. Consequently, patients who test positive for malaria are expected to be prescribed any of three recommended oral artemisinin-based combination therapy (ACT), which are artesunate–amodiaquine (ASAQ), artemether–lumefantrine (AL) or dihydroartemisinin–piperaquine (DHAP) [8], and anti-malarial treatment must generally be withheld from febrile patients who test negative for malaria parasites.

So far, prescription of ACT to patients with malaria in Ghana is high, an observation which is consistent with global trends [5]. Data from cross-sectional surveys also indicate that between 90 and 100% of patients with positive malaria tests results are prescribed appropriate treatment [9, 10]. The challenge with the implementation of the T3 policy has mainly been the failure to test suspected malaria cases in the absence of RDT stock-outs, continuance of presumptive treatment and prescription of ACT to patients who test negative for malaria (over-treatment). These practices have been documented in other malaria-endemic settings [11–13]. The proportion of patients treated presumptively (patients prescribed treatments without tests) remains high, at almost 50.0% of malaria cases [4].

Other deviations from policy recommendations, such as the prescription of anti-malarial injections and the prescription of recommended anti-malarials with sub-optimal dosage regimen to uncomplicated malaria cases have been reported [14–17]. In some cases, afebrile patients are treated with anti-malarials with or without malaria tests [9, 18]. Additionally, failure to prescribe ACT to patients who test positive for malaria [19] has been reported. Prompt and effective case management of malaria cases will prevent uncomplicated malaria cases from progressing to severe malaria, with its associated mortality as well as to ensure effective clearance of malarial parasites, which will eventually contribute to the reduction in the human reservoir host needed to facilitate malaria transmission [20]. On the other hand, inappropriately prescribed ACT leads to over-prescription of ACT, waste of ACT and laboratory diagnostics. All of these are costly to patients, their families and to malaria control programmes and their partners [11]. There is a potentially dangerous delay in treatment of the actual cause of febrile illnesses, and this can affect productivity [21, 22]. In the long run, patients may lose confidence in ACT and RDTs [12, 23, 24] and anti-malarial drug pressure could build up, accelerating the development of ACT resistance [25].

A number of observational studies conducted in Ghana, which were studying prescriber prescription practices for malaria treatment have focused on malaria meso- or hyper-endemic settings in the country. The few studies that have focused on hypo-endemic settings in the country have excluded the Greater Accra region in particular, or described anti-malarial prescribing trends, leaving some key indicators of adherence to malaria treatment guidelines [9, 26–29]. However, at community level, febrile children seen in hypo-endemic settings, such as the Greater Accra region (where the prevalence of malaria is below 5%), are more likely to be suffering from a non-malarial febrile illness compared to febrile children seen in hyper-endemic settings [30]. Findings from a hospital-based study indicates that the relative contribution of malaria (11.2%) to febrile illnesses is significantly lower than reported in routine health facility data [31]. The need for prescribers to request for malaria tests and rely on test results to aid treatment practices in hypo-endemic malaria settings is even greater. To satisfy this need, this study was designed to assess prescribers’ treatment practices for febrile patients attending public health delivery facilities in the Greater Accra Region, and to investigate the factors associated with observed inappropriate prescription of ACT.

## Methods

### Study type and location

This is a health facility-based, cross-sectional analytic study conducted in the Greater Accra region (GAR). There are 16 administrative units in the region. Six health facilities in three municipalities (Ga South, La Dade-Kotopon, La-Nkwantanang Madina) of the region were involved. The three municipalities were selected from a list of 9 administrative units with the proportion of confirmed malaria cases not greater than 24.9% per GAR’s Health Management Information System (HMIS) between January and December, 2014.

For each municipality, the two facilities that contributed the most to total OPD visits in the public health delivery sector were selected. These facilities had no recorded stock-outs of all three recommended ACT nor inability to perform malaria tests within 6 months prior to data collection. They included Kekele Polyclinic and Pentecost Hospital from the La-Nkwantanang Madina municipality; La General Hospital and Police Hospital from the La Dade-Kotopon Municipality and Ga South Municipal Hospital and Ngleshie Amamfrom Health Centre from the Ga South Municipality.

Following the recruitment of field staff and pre-testing of data collection tools at the Ashaiman Polyclinic, located in Ashaiman Municipality, a health facility survey within the selected study municipalities was conducted between October and December 2015. Excluding the records of review patients, afebrile patients, pregnant women and febrile patients with danger signs, all other records of febrile OPD patients who had visited these health facilities were obtained from the records department. OPD folders of patients who had axillary temperature (T), T ≥ 37.5 °C or fever recorded in clinical notes were classified as febrile.

The improved data extraction tools (following the pre-test) were used to retrieve information on patient age, gender and ownership of a health insurance card, presenting symptoms, laboratory investigations conducted, laboratory results, diagnoses, and prescribed drugs. Malaria diagnoses documented in OPD records of patients encompassed both confirmed and suspected malaria cases. Other diagnoses may not have been based strictly on standard International Classification of Diseases (ICD) codes.

Interviews were held with the prescribers who saw these patients in order to obtain information on prescriber demographic characteristics, professional category, work experience, exposure to in-service training on Integrated Management of Neonatal and Childhood Illnesses (IMNCI), Malaria Case Management (MCM), access to MCM guidelines and wall-charts and supervision. Assessment tools were employed to determine availability of microscopy, RDT services and anti-malarials in laboratories and pharmacies of each health facility.

Since the study mostly used routinely collected data, potential inconsistency in the measurement of temperature due to differences in thermometers used and inter-operator variability, use of different brands of RDT, differences in the efficiency of different microscopes used at the different hospitals, and between prescriber variabilities that were not measured, may have had an impact on the findings of this study.

### Data management and analysis

Epidata version 3.1 and Stata version 13 SE (College Station, TX, USA) were used to manage and analyse the data. The data were checked to ensure internal consistency and completeness. The analyses were restricted to the initial visits of febrile patients who were at least 6 months or older who did not present with any danger signs or severe disease. The data obtained from prescribers, patient records, facility pharmacies, and laboratories were merged into one dataset using identifiers for municipalities, facilities and prescribers. The number of febrile cases who were attended by a prescriber in a facility on a survey day constituted a cluster. On the basis of this, the data was “svyset” and analyses were conducted, adjusting for clustering. The characteristics of the prescribers and the patients seen were described using simple frequencies. For continuous variables such as age and prescriber number of years of work, means and standard deviations were used to summarize them after log transformations of continuous variables that were skewed. For categorical variables, Chi square tests were used to determine differences.

The primary outcome investigated was inappropriate prescription of ACT, a composite binary indicator which combined the proportion of febrile patients who were either presumptively treated or over-treated. Febrile patients with negative malaria test results who were prescribed ACT were considered to have been over-treated, whilst febrile patients who were not tested but prescribed ACT were considered to have been treated presumptively. Combining these two proportions provided a good measure of the fraction of ACT prescribed inappropriately. Explanatory variables included patient level factors (age-group and ownership of a health insurance card), prescriber level factors and health facility level factors (availability of microscopy services, RDTs and anti-malarials). The crude association between the outcome variable and all other predictor variables were examined using Odds Ratios. A logistic regression analysis was performed to determine the predictors of the primary outcome at 95% CI (α-value of 0.05). The model comprised the outcome, a variance covariate estimator (vce) with the clustering variable and all independent variables that were predictors of the outcome at p < 0.1 in the crude analysis.

## Results

### Descriptive characteristics

Overall, the records of 2519 independent febrile OPD patients were analysed. Of this number of patients, 45.6% (n = 1149) were younger than 5 years and 1.2% (n = 30) had no record of age nor date of birth on their folders. Their ages ranged from 6 months to 99 years, with a mean age of 6.8 years and a standard deviation of 4.4 years. Females constituted 52.7% (1327) of patients. Additionally, 57.6% of these patients were registrants of the National Health Insurance Scheme (NHIS) or other health insurance schemes. These patients were seen by 82 prescribers, whose mean age was 32.2 years with a standard deviation (SD) of ± 1.28, and the distribution showed that 80.2% of them were below the age of 40 years. Females constituted 61.0% of the prescribers. Medical officers and physician assistants constituted 59.8 and 29.3% of the prescribers, respectively. The mean number of years of work experience for the prescribers was 3.4 years (SD ± 2.6).

### Prescriber-patient interactions

A little over half of all the patients (51.6%) were attended by physician assistants. Medical officers saw 1065 (42.3%) patients whilst the remaining 153 were seen by nurses and student prescribers. During these interactions, as many as 1512 (60.0%) of the patients were not referred for malaria tests and the rest [40.0% (95% CI 37.1–43.0%)] were tested by microscopy or RDTs.

**Fig. 1:**
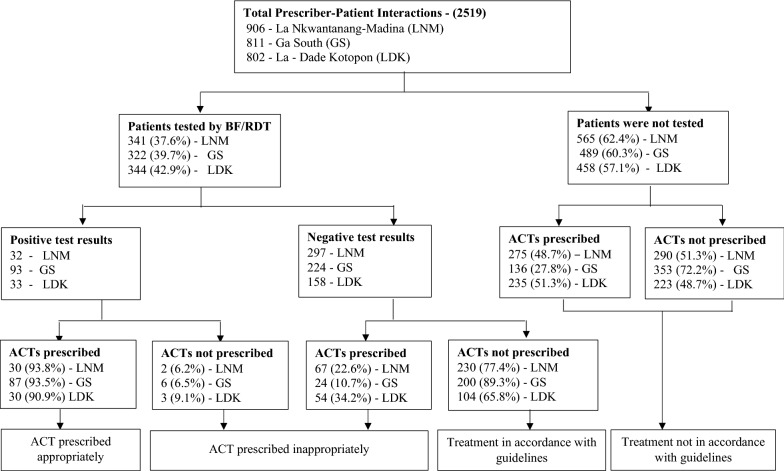
Malaria case management practices of prescribers in the three municipalities

Overall, as high as 98.0% of the patients were prescribed drugs, with a mean number of drugs per prescription of 3.2 (SD ± 1.2). Recommended oral ACT was prescribed for 1036 (41.1%) patients, whilst 234 patients were prescribed artemisinin monotherapy injections. However, 19.4% of the patients (n = 201) who were prescribed oral ACT were also prescribed artemisinin monotherapy injections concurrently. On the average, out of every 5 febrile patients prescribed ACT, 4 were also prescribed another drug in addition to the ACT; 83.4% were prescribed antipyretics, 61.4% were prescribed antibiotics and 10.4% (108 of 1036) were prescribed haematinics. None of the OPD folders sampled contained prescriptions of non-artemisinin monotherapy, including quinine and amodiaquine. Figure 1 provides detail of the malaria case management practices of the prescribers at each municipality.

In respect of the relationship between failure to test and prescription, almost half (42.7%: 95% CI 38.8–46.7) of the 1512 patients who were not tested were prescribed ACT. Among the 1007 patients who were tested, 158 tested positive for malaria, of which 147 (93.0%; 95% CI 87.6–96.2%) were prescribed recommended ACT. The remaining 11 patients with confirmed malaria were neither prescribed recommended nor non-recommended anti-malarials. Seven out of these 11 patients were however prescribed antibiotics. On the other hand, of the 679 patients who had negative malaria tests results, 145 (21.4%; 95% CI 17.9–25.3) were prescribed ACT.

**Fig. 2:**
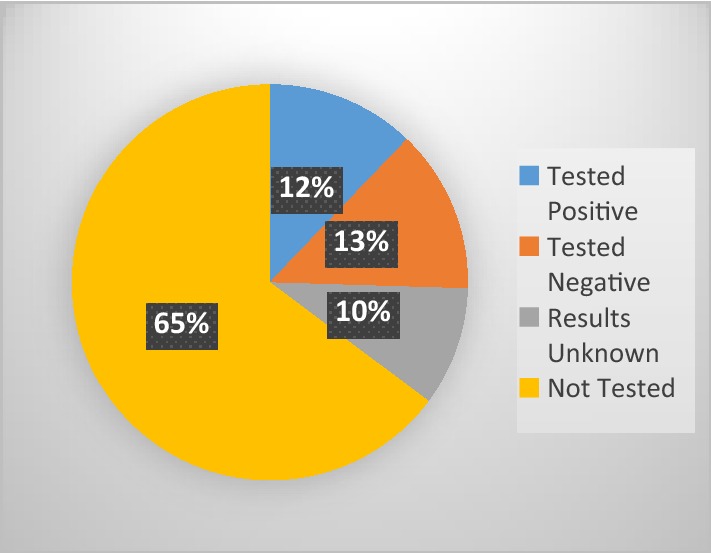
ACT prescription by malaria confirmatory tests. The figure presents the proportion of ACT shared among patients. Patients who were not tested benefited from more than half of total ACT prescriptions

In all, the proportion of patients who were prescribed ACT inappropriately was 76.4% (n = 791 of 1036), (95% CI 73.6–78.9). Of these 791 patients, 141 (17.8%) patients were prescribed artemisinin monotherapy injections in addition. The fractions of ACT prescriptions based on malaria test results are highlighted in Fig. 2.

The association of patient characteristics and treatment showed that under-fives were significantly less likely (χ^2^ = 7.0388, p < 0.04) to be tested for malaria 37.2% (95% CI 33.5–41.0%), compared to those who were 5 years and older 42.4% (95% CI 39.0–45.9%). Additionally, patients younger than 5 years were significantly less likely (χ^2^ = 6.5045, p < 0.05) to be treated presumptively (39.2%, 95% CI 33.9–44.8) compared to patients who were 5 years and older (45.7%, 95% CI 41.3–50.2). Significantly fewer (χ^2^ = 6.7472, p < 0.05) under-fives were overtreated (17.2%: 95% CI 13.1–22.4%) compared to those above 5 years, 25.5% (95% CI 20.6–31.0%), Over a third (36.8%) of the under-fives were diagnosed with malaria compared to 44.1% of patients above 5 years of age, (χ^2^ = 13.6163, p < 0.01). There was no significant difference (χ^2^ = 0.1827, p > 0.05) in the prescription of ACT to patients with positive test results by age-group, 94.1% of under-fives compared to 92.2% of patients above 5 years. The top ten symptoms and diagnoses of the patients are presented in Table 1.

**Table 1:** Top ten symptoms and diagnoses of febrile patients

Symptom	Frequency	Percent	Diagnosis^a^	Frequency	Percent
Headache	1258	49.9	ARI	1106	43.9
Poor appetite	1120	44.5	Malaria	1029	40.8
Nasal congestion	1032	41.0	UTI	819	32.5
General body pains	995	39.5	Typhoid	772	30.6
Diarrhoea	860	34.1	Enteritis	766	30.4
Dizziness	841	33.4	Pneumonia	732	29.1
Cough	828	32.9	Ear infection	731	29.0
Fatigue	804	31.9	Tonsillitis	726	28.8
Pain on passing urine	784	31.1	Anaemia	715	28.4
Nausea	747	29.7	Eye infection	702	27.9
Sample size	2519		Sample size	2519	

^a^Diagnoses may not be based on standard ICD codes

### Factors associated with inappropriate ACT prescription

In a crude analysis (Tables 2 and 3), a number of variables were significantly associated with inappropriate ACT prescription, and these included, age-group of patients, treatment chart availability, access to reference material on Malaria Case Management (MCM) and training in IMNCI or MCM in the past 12 months. However, after adjusting for covariates, prescriber factors, such as number of training programmes in MCM or MCM attendance, number of supervisory visits on MCM, access to ACT treatment charts or wall charts on MCM, work experience, professional category and RDT stock-outs became significantly associated with inappropriate ACT prescription.

**Table 2:** Regression analysis for the association between inappropriate ACT prescription and non-prescriber covariates

Variable	Crude OR (95% CI)	p-value	AOR (95% CI)	p-value
Facility level factors				
RDT stock-out				
Yes^a^	1		1	
No	0.55 (0.36–0.85)	0.007	0.04 (0.01–0.14)	< 0.001
Municipal				
Ga South^a^	1		1	
La Dade Kotopon	1.3 (0.77–2.19)	0.322	Omitted	
La Nkwantanang Madina	6.4 (3.2–12.8)	< 0.01	0.37 (0.10–1.29)	0.117
Patient level factors				
Patient age-group				
<5^a^	1		1	
≥5	0.61 (0.4–0.9)	0.004	0.80 (0.54–1.19)	0.265
Access to NHIS				
No^a^	1			
Yes	0.85 (0.62–1.2)	0.329		

*AOR* adjusted odds ratio, *OR* odds ratio

^a^Reference category/group

**Table 3:** Regression analysis for the association between inappropriate ACT prescription and prescriber-related factors

Prescriber level factors	Crude OR (95% CI)	p-value	AOR (95% CI)	p-value
Prescriber work experience (years)				
< 3 years^a^	1		1	
3 to less than 6 years	0.96 (0.6–1.6)	0.871	0.84 (0.45–1.6)	0.568
≥ 6 years to < 10 years	0.5 (0.25–1.0)	0.054	0.4 (0.21–0.74)	0.004
≥ 10 years	3.4 (1.7–6.8)	0.001	5 (2.4–10.4)	< 0.001
Professional category				
Physician assistants^a^	1		1	
Doctors	0.48 (0.28–0.80)	0.005	5.8 (2.5–13.7)	< 0.001
Others	1.73 (0.08–0.38)	< 0.001	0.1 (0.03–0.27)	< 0.001
Supervision in past 6 months				
No^a^	1			
Yes	0.77 (0.5–1.2)	0.245		
Number of MCM supervisory visits				
0^a^				
Once	1.4 (0.77–2.5)	0.271	8.2 (3.3–20.6)	< 0.001
2–3 times	0.47 (0.28–0.77)	0.003	1.0 (0.44–2.12)	0.944
> 4 times	0.74 (0.43–1.24)	0.284	1.4 (0.79–2.50)	0.246
Supervision on MCM in past 6 months				
No^a^	1			
Yes	0.86 (0.45–1.66)	0.653		
Access to any reference material on MCM				
No^a^	1		1	
Yes	1.6 (1.1–2.4)	0.022	1.7 (0.95–3.0)	0.075
Access to revised 2014 guideline				
No^a^	1			
Yes	1.37 (0.80–2.3)	0.25		
ACT treatment chart availability				
No^a^	1		1	
Yes	0.59 (0.4–0.9)	0.021	9.0 (3.7–22.2)	< 0.001
Availability of MCM wall chart				
No^a^	1		1	
Yes	0.39 (0.26–0.59)	< 0.001	0.12 (0.06–0.24)	< 0.001
Visibility of wall charts				
No^a^	1			
Yes	0.39 (0.26–0.59)	< 0.001	Omitted	
Training in MCM in past 12 months				
No^a^	1		1	
Yes	3.5 (2.3–5.2)	< 0.001	0.95 (0.33–2.7)	0.922
Number of IMNCI training in past 12 months				
0^a^	1		1	
Only 1	0.25 (0.15–0.44)	< 0.001	0.07 (0.03–0.15)	< 0.001
2 to 3	2.3 (0.34–15.9)	0.389	2.8 (0.31–25.6)	0.36
4 and above	1.55 (0.16–15.2)	0.706	2 (0.19–20.7)	0.563
Number of MCM training in past 12 months				
0^a^	1		1	
Only 1	3.4 (2.2–5.3)	< 0.001	4.1(1.5–11.6)	0.007
2 to 3	4.2 (2.1–8.5)	< 0.001	Omitted	
4 and above	0.6 (0.14–2.3)	0.429		
Training in IMNCI in past 12 months				
No^a^			1	
Yes	0.31 (0.18–0.53)	0.001	Omitted	
Ever trained in MCM				
No^a^	1		1	
Yes	1.8 (1.1–3.0)	0.016	0.97 (0.47–2.02)	0.94

*AOR* adjusted odds ratio, *OR* odds ratio

^a^Reference category/group

Specifically, febrile patients who were seen in health facilities with RDTs in stock were less likely to be prescribed ACT inappropriately, (AOR: 0.04, 95% CI 0.01–0.14, p < 0.001) compared to those who were seen in health facilities with RDT stock-outs. Patients who were seen by medical officers were more likely to be prescribed ACT inappropriately than the patients seen by physician assistants (AOR: 5.8, 95% CI 2.5–13.7, p < 0.001). The odds of patients being prescribed ACT inappropriately by student and nurse prescribers was reduced by 90% compared to those seen by physician assistants (AOR: 0.1, 95% CI 0.03–0.27, p < 0.001). Patients who were seen by prescribers who had been supervised once within the past 6 months were 8 times more likely to be prescribed ACT inappropriately compared those seen by prescribers who had not benefited from any supervision on MCM within the past 6 months. The odds of patients being prescribed ACT inappropriately by prescribers who had access to MCM wall charts was reduced by more than half (88%) compared to the prescribers who did not have the MCM wall chart (AOR: 0.12, 95% CI 0.06–0.24, p < 0.001). However, the odds of patients being prescribed ACT inappropriately by prescribers who had access to the ACT treatment chart was increased by 800% compared to the prescribers who did not have the ACT treatment chart (AOR: 9.0, 95% CI 3.7–22.2, p < 0.001).

Prescribers who had benefited from in-service training intervention on malaria case management at least once within the past year were 4 times more likely to prescribe ACT inappropriately compared to those who had not benefited from any MCM training intervention in the past year (AOR: 4.1; 95% CI (1.5–11.6); p < 0.01). However, the odds of patients being prescribed ACT inappropriately by prescribers who had been trained at least once in IMNCI within the past 12 months was reduced by 93% compared to the prescribers who had not been trained IMNCI the past year (AOR: 0.07, 95% CI (0.03–0.15), p < 0.001). In this study, over 70% (1774) of patients were prescribed antibiotics including 45.6% (72) of febrile patients with confirmed malaria and 70.4% (478) of patients with negative malaria test results.

## Discussion

Managing malaria cases effectively at health facility level depends on effective case detection through systematic screening of febrile cases and prompt treatment with effective anti-malarials. In the effort to assess the extent to which these occur in the Greater Accra region of Ghana, it was observed that less than half (40.0%) of the febrile cases seen were screened for malaria, resulting in lost opportunities of malaria screening for majority of febrile cases, a requirement of the T3 policy [20].The low testing rates observed appears to be consistent with estimates of earlier studies in Ghana where testing rates much lower than 40.0% were observed [9, 32].

One out of five patients with negative test results was over-treated with ACT whilst 43 out of every 100 patients who were not tested for malaria were treated presumptively leading to a high fraction of ACT prescribed inappropriately (Fig. 1), and prescription of artemisinin monotherapy injections alongside is rather worrying. Elsewhere, a high fraction of inappropriate ACT prescription was observed during the pre-intervention phase of a quasi-experiment in Namibia [33]. In Ghana, Orish et al. [27] reported from a study among children (0–12 years) at the OPD of a paediatric regional hospital in the Western Region, that 84.1% of patients without malaria and 78.2% of the children who were not screened for malaria were treated with anti-malarials. Their results appear to be higher than estimates from the current study. This could possibly be attributed to the fact that the study population were children, not older than 12 years. In another study in Ghana, with even younger study participants (under 5 years old) however, the point estimate for over-treatment was 21.9%, which is similar to the present research findings. Since the study sites were chosen from a list of administrative units with poor case management practices as of September 2014 (HMIS), it is plausible that these practices persisted until the time of data collection of the current study.

Such high rates of inappropriately prescribed ACT can potentially erode the reduction in morbidity and mortality associated with the introduction of ACT if effective interventions are not introduced to curb the trend. In a hospital-based survey in the Police Hospital, 2036 (30.4%) OPD patients were prescribed anti-malarial drugs and anti-malarial injections [26]. Inappropriately prescribed oral ACT and prescription of parenteral artemisinin injections, which is reserved for the treatment of severe malaria, to OPD febrile cases will increase drug expenditure and produce poor heath eventually [20]. The use of injections can be beneficial to the patient if prescribed and administered appropriately but can be equally risky if caution is not taken. Lack of public education, scarce expertise and patient belief and demands are causes of injection abuse. In many countries there is the belief among patients and prescribers that injections work faster and have a stronger effect than oral medications. Moreover, they come with higher charges than the oral forms of anti-malarials. Regulating the use of injections is necessary as it reduces the risk of complications in the form of abscesses at injection site, unbearable pains from needle prick, disabilities and possible exposure to infections when unsterilized needles are used [15, 16, 34, 35].

Although not all the patients who needed ACT were prescribed (Fig. 1), the observed prescription of ACT to patients with confirmed malaria was remarkably high (over 90%). This observation is comparable to findings from other health facility surveys that reported rates above 90% [14, 15, 36). Regarding failure to prescribe ACT to patients with confirmed malaria, ACT stock-outs may account for this phenomenon [37]. In the current study however, this was not the case because all study sites had stocks of at least 2 recommended ACT during the data collection period. Further research is needed to understand why ACT are not prescribed although these are available, given that delay in treatment of a malarial infection with ACT, especially in vulnerable groups such as under-fives can result in severe disease and deaths.

Among the factors associated with malaria case management practices of prescribers are prescriber, patient and health facility factors. Regarding patient factors, since patients who own health insurance cards are less likely to face challenges with paying for the costs of their malaria tests and treatments, it is not unreasonable to expect such patients to be prescribed ACT more appropriately. However, ownership of a valid health insurance card by over half of the febrile patients appeared not to impact on prescriber management practices for febrile illnesses even in the crude analysis. Two other cross-sectional studies in Ghana, in which between 88.6 and 92.0% of study participants owned health insurance cards, reported a lack of effect of valid NHIS cards on quality of MCM [9, 28].

Concerning health facility factors, malaria diagnostics are required for malaria tests and their role in reducing inappropriately prescribed ACT was observed in this study (Table 2), considering the reduced odds (by 96.0%) of being prescribed ACT inappropriately among patients seen in facilities that had RDTs in stock. Similar observations were made by Bawate et al. [38] in a health facility cross-sectional survey in Uganda. Steps must be taken to consciously increase the proportion of febrile cases benefiting from malaria testing through reliable RTD supplies to health facilities alongside reducing the waiting time associated with requesting and waiting for malaria test results by moving RDTs for outpatients systematically from laboratories to OPDs in hospitals [39].

In respect of prescriber factors, among medical officers, physician assistants and nurse prescribers, lower cadre staff were found to be less likely to prescribe ACT inappropriately compared to higher cadre staff. This observation has been established in a number of cross-sectional studies [9,19, 40, 41]. Kwarteng et al. [9] reported that lower cadre staff who work in health centres and Community-Based Health Planning and Services compounds were likely to adhere to guidelines compared to higher cadre staff who work in hospitals in the middle belt of Ghana. Prescribers with more than 10 years’ work experience were more likely to prescribe ACT inappropriately compared to those with fewer years work experience. Selemene et al. [41] observed that health workers with 3 or more years work experience were more likely to adhere to malaria treatment guidelines, as a result of possibly witnessing the direct effects of adhering to treatment guidelines over their many years of practice.

To guarantee effective MCM, sick patients should be able to access care in facilities equipped with basic diagnostics and drugs for effective case management; health workers at post must be equipped with the right knowledge and skills to effectively manage patients. The NMCP of Ghana coordinates, trains and supervises health workers and the supply of malaria commodities, guidelines and wall charts on case management to health facilities. Unfortunately, many other studies have concluded that the direct effect of these interventions at improving MCM practices has not always been consistent [9, 38, 40, 42]. The research findings suggest that training in MCM within the past year, supervision within the past 6 months and access to ACT treatment charts in particular may not necesary lead to a reduction in inappropriate prescription of ACT. Smith et al. [43] in a systematic review involving more than 20 studies observed that most cross-sectional studies have reported a lack of effect of interventions introduced at the health facility level to improve case management of malarial cases.

Deploying quality improvement tools during supervision of health workers and relying on training strategies that are less didactic, less formal and more on-the-job training could improve expected gains post training [43, 44]. In addition, qualitative and quantitative evaluation of training interventions in the country may potentially provide useful insights to improve upon training interventions programme implementation in the country.

Of concern is the concomitant prescription of antibiotics to febrile outpatients with or without malaria. The prevalence of antibiotic prescription for febrile patients was observed to be approximately 40% higher than the 50% estimated prevalence among the general population of patients in sub-Saharan Africa [45]. This finding is consistent with the recent reported increases in prescribing of antibiotics globally, especially in African countries [45–47]. The need for MCM training interventions that emphasizes the rational use of antibiotics in the treatment of non-malarial illnesses will go a long way to reduce waste of resources, antibiotic resistance and delay artemisinin resistance simultaneously [23].

### Study limitations

To obtain data on febrile outpatients in this study, the investigators relied on secondary data alone without a gold standard, implying that any potential misclassifications could not be accounted for. Findings of the study should therefore be interpreted with caution. The data used to arrive at these conclusions is cross-sectional in nature and the observed temporal associations between exposure and outcome variables may not be causal.

## Conclusions

Inappropriate ACT prescription to OPD febrile cases was observed to be cross-sectionally and unacceptably high. Proven interventions such as training and supervision of health workers and supply of guidelines on case management appear not to be yielding the desired outcomes. Reducing stock-outs of RDTs can potentially improve case management. Health authorities could pay more attention to high cadre staff and those with many years of experience during planning and implementation of interventions to improve malaria case management at the health facility level.

## Data Availability

The datasets used and/or analysed during the current study are available from the corresponding author on reasonable request.

## Abbreviations

ACT: artemisinin-based combination therapy
AL: artemether–lumefantrine
ASAQ: artesunate–amodiaquine
BF: blood film for malaria parasite
DHAP: dihydroartemisinin–piperaquine
GAR: Greater Accra Region
HMIS: Health Management Information System
ICD: International Classification of Diseases
IMNCI: Integrated Management of Neonatal and Childhood Illnesses
MCM: Malaria Case Management
NHIS: National Health Insurance Scheme
NMCP: National Malaria Control Programme
OPD: outpatient department
RDT: rapid diagnostic test
T3: Test, Treat and Track
WHO: World Health Organization
